# High-Performance X-Ray Detection and Optical Information Storage via Dual-Mode Luminescent Modulation in Na_3_KMg_7_(PO_4_)_6_:Eu

**DOI:** 10.3390/molecules30173495

**Published:** 2025-08-26

**Authors:** Yanshuo Han, Yucheng Li, Xue Yang, Yibo Hu, Yuandong Ning, Meng Gu, Guibin Zhai, Sihan Yang, Jingkun Chen, Naixin Li, Kuan Ren, Jingtai Zhao, Qianli Li

**Affiliations:** 1 State Key Laboratory of Materials for Advanced Nuclear Energy, School of Materials Science and Engineering, Shanghai University, Shanghai 200444, China; hys13403872261@163.com (Y.H.); sweenamyysh@shu.edu.cn (S.Y.);; 2 Shanghai Frontier Base of Intelligent Optoelectronics and Perception, Institute of Optoelectronics, Fudan University, Shanghai 200433, China; liyc24@m.fudan.edu.cn; 3 Guangxi Key Laboratory of Information Materials, Guilin University of Electronic Technology, Guilin 541004, China; 4 Laser Fusion Research Center, China Academy of Engineering Physics, Mianyang 621050, China

**Keywords:** Na_3_KMg_7_(PO_4_)_6_:Eu, photochromism, radio-photoluminescence, dual-mode luminescent, X-ray imaging

## Abstract

Lanthanide-doped inorganic luminescent materials have been extensively studied and applied in X-ray detection and imaging, anti-counterfeiting, and optical information storage. However, many reported rare-earth-based luminescent materials show only single-mode optical responses, which limits their applications in complex scenarios. Here, we report a novel Na_3_KMg_7_(PO_4_)_6_:Eu phosphor synthesized by a simple high-temperature solid-state method. The multi-color luminescence of Eu^2+^ and Eu^3+^ ions in a single matrix of Na_3_KMg_7_(PO_4_)_6_:Eu, known as radio-photoluminescence, is achieved through X-ray-induced ion reduction. It demonstrated a good linear response (R^2^ = 0.9897) and stable signal storage (storage days > 50 days) over a wide range of X-ray doses (maximum dose > 200 Gy). In addition, after X-ray irradiation, this material exhibits photochromic properties ranging from white to brown in a bright field and shows remarkable bleaching and recovery capabilities under 254 nm ultraviolet light or thermal stimulation. This dual-modal luminescent phosphor Na_3_KMg_7_(PO_4_)_6_:Eu, which combines photochromism and radio-photoluminescence, presents a dual-mode X-ray detection and imaging strategy and offers a comprehensive and novel solution for applications in anti-counterfeiting and optical information encryption.

## 1. Introduction

The high energy and strong penetration of X-rays enable them to play an irreplaceable role in medical diagnosis, non-destructive testing, and environmental monitoring [1,2,3,4]. However, with the increasing complexity and diversity of application scenarios for X-ray detection and imaging technology, the demand for high-performance X-ray detection and imaging—featuring high efficiency, stability, and precision—continues to grow [4,5]. Currently, X-ray detection and imaging mainly use two approaches. One involves semiconductors that directly convert X-ray energy into charges, effectively reducing signal loss and improving time and spatial resolution [6,7]. The other uses scintillators to convert X-ray energy into ultraviolet or visible light, then converts it to electrical signals through photoelectric devices such as charge-coupled devices (CCDs). The advantage of indirect conversion lies in its relatively mature process and lower cost, though it requires extra light conversion steps, potentially introducing signal distortion and noise [3,8,9].

These technologies have their own limitations. Direct conversion using semiconductor materials such as CdZnTe and HgI_2_ faces challenges including uneven charge transport, significant noise, and high operating voltage [10,11]. Indirect conversion using scintillation materials also has drawbacks. Inorganic scintillators such as (Y, Lu)_2_SiO_5_ (LYSO) and Ce-doped Gd_3_(Al, Ga)_5_O_12_ (GAGG), are difficult to prepare and prone to defects, while metal halide perovskite scintillators can decompose under humid, high-temperature, or high-energy radiation conditions [1,3,12]. Organic scintillators have been widely studied and applied due to their significant advantages in processability and cost, but issues such as non-radiative decay and poor stability still need to be addressed [13,14]. Furthermore, traditional scintillation materials are widely used in two-dimensional planar imaging, especially for real-time monitoring. However, due to the limitations of physical properties, stability, and processing, these materials are difficult to meet the flexibility and diversity requirements of X-ray detection and imaging technologies, such as simultaneous flexible imaging and evaluation of long-term accumulated X-ray radiation doses [8,15]. The development of X-ray persistent luminescent materials (XPEL) has improved these limitations to some extent [16]. These materials can store energy excited by X-rays and continuously release it as photons after the radiation source is removed, as in NaLuF_4_:Tb@NaYF_4_ and Tb^3+^@NaMgF_3_ [16,17,18]. Their rational design significantly extends the afterglow lifetime, but their inherent time-dependent decay characteristics still limit signal stability and reusability [19,20].

Some inorganic phosphors exhibit reversible changes in their optical properties under alternating stimulation by X-ray and UV-Vis photons or thermal treatment, offering new possibilities for X-ray detection and imaging [21,22,23]. The photochromic effect and radio-photoluminescence are influenced by radiation dose and time, demonstrating excellent signal stability and reusability, thus breaking through traditional limitations [24,25,26,27,28]. Integrating X-ray-induced photochromism and radio-photoluminescence into a single material is a promising strategy for multifunctional applications, with significant potential in dose monitoring, anti-counterfeiting, medical imaging, and security inspection [23,29]. However, these composite materials often suffer from low luminescence efficiency due to performance mismatch and physical–chemical incompatibility [30,31]. Therefore, researchers have shown great interest in developing new materials capable of multi-mode luminescence and high X-ray detection capability.

To achieve X-ray-induced multi-mode luminescence in a single material, trivalent lanthanide-activated inorganic phosphors have attracted extensive attention due to their unique properties, including rich 4f energy levels, characteristic sharp line emissions, and significant effects even with trace doping [21,32]. Particularly, Eu^3+^ have been extensively studied in the field of high-performance luminescence due to their high color purity, excellent emission wavelength stability and thermal stability. Additionally, selecting an appropriate host material is crucial for achieving optimal optical performance [33]. Considering the hydrolysis of silicate [34,35], high sintering temperature and high-cost of zirconate [36,37], and inadequate stability under X-ray of borate [38,39], phosphate hosts emerge as a good choice due to their low-cost, facile processing, and exceptional thermal stability [40,41,42]. Recently, Pan et al. successfully synthesized isomorphic Na_3_AMg_7_(PO_4_)_6_ (A = K and Cs) with flexible frameworks and large band gaps [43]. The large bandgap of the host can effectively suppress thermal ionization of 5d excited-state electrons, which is crucial for developing radio-photoluminescence phosphors. A zero-thermal-quenching Na_3_KMg_7_(PO_4_)_6_:Eu^2+^ blue phosphor has been reported, featuring high quantum efficiency, excellent chemical stability, and color stability [44,45]. Meanwhile, another study pointed out that Eu^3+^-doped Na_3_KMg_7_(PO_4_)_6_ phosphor has excellent thermal stability and remarkable luminescence performance [46]. Inspired by these works, this study investigates Eu^3+^-doped Na_3_KMg_7_(PO_4_)_6_ and explores its photochromic and radio-photoluminescence properties induced by X-rays.

The color-changing process of Na_3_KMg_7_(PO_4_)_6_:Eu (NKMPO:Eu) under X-ray irradiation and the bleaching process under 254 nm ultraviolet light (UV) or thermal stimulation were analyzed. A linear relationship was found between the degree of photochromism and X-ray dose. Its stability and reversibility were evaluated. Meanwhile, the radio-photoluminescence phenomenon was studied in detail, and dose and time-dependent changes under X-ray irradiation were systematically explored. NKMPO:Eu demonstrated an excellent high-dose X-ray linear response, long-term signal storage capability, and reusability, highlighting its potential as an X-ray dosimetry and imaging material. Additionally, a model of the interaction between X-rays and NKMPO:Eu was proposed to discuss the generation and relationship of photochromism and radio-photoluminescence effects. Finally, by combining NKMPO:Eu powder with room-temperature vulcanized silicone rubber (RTV) to fabricate flexible films, a dual-mode X-ray detection and imaging method was developed, enabling applications such as information encryption and anti-counterfeiting. NKMPO:Eu integrates photochromism and radio-photoluminescence in a single material, achieving multi-mode X-ray detection and imaging, providing a feasible approach for flexible X-ray detection and storage with high information stability and easy readout, as well as for anti-counterfeiting and information encryption applications.

## 2. Results and Discussion

### 2.1. Synthesis and Structure Description

**Figure 1:**
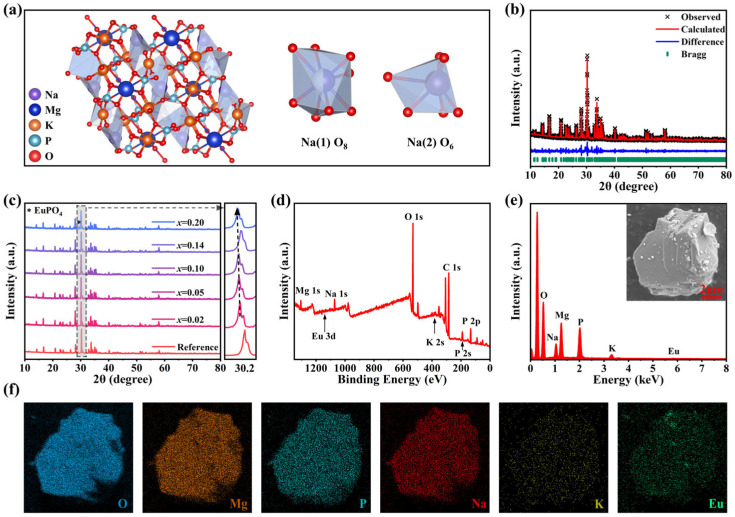
(**a**) Crystal structure of NKMPO and the illustration of two crystallographic sites for Na atoms. (**b**) XRD refinement results of NKMPO:0.14Eu. The reference XRD pattern of NKMPO crystal is from Ref. [43]. (**c**) XRD patterns of NKMPO:*x*Eu (*x =* 0.02–0.20) samples. The reference XRD pattern of NKMPO crystal is from Ref. [43]. (**d**) XPS survey spectra of the NKMPO:0.14Eu powder. (**e**) EDS spectrum, and (**f**) EDS elemental mapping of the NKMPO:0.14Eu powder.

A series of NKMPO:*x*Eu (*x* = 0.01–0.20, 1–20 mol%) powders were synthesized using a high-temperature solid-state method, and NKMPO:Eu@RTV films of various sizes were fabricated by mixing NKMPO:Eu with RTV glue in a certain proportion and then drying them. Figure S1 shows the preparation process. NKMPO crystallizes in the monoclinic space group *C*2/*c*, and its crystal structure can be divided into two parts, as shown in Figure S2. NKMPO exhibits a complex three-dimensional framework of isolated phosphates and MgO*_x_* (*x* = 5 and 6), with K and Na atoms located in channels along the [010] direction. Located within an expansive interstitial cavity, the large potassium atom is coordinated by 12 oxygen atoms. Meanwhile, Na (1) and Na (2) atoms are coordinated by eight and six O atoms, respectively, and Mg (1)–Mg (5) atoms are coordinated by five and six O atoms, respectively [43,45,47]. In the NKMPO crystal structure, Eu^3+^ is expected to be doped at Na (1) and Na (2) sites, as shown in Figure 1a. In general, ion substitution tends to achieve the lowest formation energy in the system, so minimizing the cation radius difference between Eu^3+^ and the host is conducive to inhibiting lattice distortion and promoting ion substitution. According to the calculated ion radius difference D_r_ (Table S1), it is inferred that Eu^3+^ may preferentially occupy the crystallographic site of Na^+^, with D_r_ Values corresponding to Na (1) and Na (2), which are 9.32% and 6.86%, respectively. Ionic radii were sourced from Shannon’s crystallographic compilation [48] for common coordinations and machine learning predictions for uncommon cases [49,50]. It should be noted that the valence state of Eu^3+^ differs from that of Na^+^, which may lead to lattice defects in achieving charge balance. Possible charge compensation mechanisms are:

(1) $$Eu+3Na={Eu}_{Na}^{∙∙}+2V_{Na}^{\prime }$$

During the charge compensation process, each Eu^3+^ substituting Na^+^ contributes two excess positive charges (${Eu}_{Na}^{∙∙}$), and at the same time, two negative vacancies will be formed at Na^+^ sites ($V_{Na}^{\prime }$) for charge balance.

X-ray diffraction (XRD) patterns (Figure 1c and Figure S3) show that a series of NKMPO:Eu samples synthesized with different doping concentrations, and processes are consistent with the XRD PDF card of NKMPO obtained through simulation and experimental verification in Ref. [43], confirming that single-phase NKMPO was obtained. These XRD patterns reveal that for samples doped with higher concentrations of Eu, due to the flexible framework of the material and the similar radius of the doped ions, the high doping concentration did not cause the failure of NKMPO monopase synthesis. For samples doped with higher concentrations of Eu, there is an impurity peak of EuPO_4_ near 30°. It can be inferred that the content of EuPO_4_ in NKMPO:Eu is relatively low because other characteristic peaks of EuPO_4_ are not visible. To obtain detailed crystal structure information, the XRD pattern of different doping concentrations was subjected to Rietveld refinement (Figure 1b, Table S2). Rietveld refinement confirms successful incorporation of Eu^3+^ ions at Na^+^ lattice sites without significant local distortion. However, progressive unit cell volume expansion emerges with increasing dopant concentration, consistent with the diffraction peak’s shift toward lower angles observed in Figure 1c. This phenomenon originates from charge-compensation-dominated lattice distortion: although substitution of Na^+^ sites (CN = 6, r = 1.02 Å; CN = 8, r = 1.18 Å) by smaller Eu^3+^ ions (CN = 6, r = 0.95 Å; CN = 8, r = 1.07 Å) would normally induce lattice contraction, the formation of sodium vacancies ($V_{Na}^{\prime }$) for charge balance triggers substantial local lattice relaxation [46]. The resulting expansion effect exceeds the contraction caused by ionic size mismatch [44]. Meanwhile, the XPS survey spectra and Energy Dispersive Spectroscopy (EDS) elemental analysis indicate that Eu is successfully doped into the NKMPO crystal structure, and Na, K, Mg, P, O, and Eu are uniformly dispersed in the selected particles (Figure 1d–f).

### 2.2. Radio-Photoluminescence Property

To evaluate the maximum X-ray detection dose of NKMPO:Eu, a series of concentration-doped NKMPO:Eu were synthesized, and the effects of Eu^3+^ doping content and synthesis conditions on the luminescence intensity was investigated. Figure S4 shows the PL spectra of NKMPO:0.02Eu under different synthesis temperature conditions. Between 1073 K and 1123 K, the PL intensity increases with synthesis temperature. This effect is attributed to the fact that higher synthesis temperatures facilitate the integration of Eu^3+^ into the NKMPO lattice, replacing Na^+^ ions and promoting better crystal quality. Figure S5 presents PL spectra and PL intensity at 612 nm of NKMPO:*x*Eu (*x* = 0.02, 0.05, 0.10, 0.13, 0.14, 0.15, 0.18, and 0.20) in the absence of X-ray irradiation. The PL intensity increases with Eu concentration up to 0.14, after which it decreases due to concentration quenching, which raises the probability of non-radiative transitions. When the doping concentration reaches 0.20, the PL intensity decreases significantly. At the same time, the XRD results show that the intensity of the impurity peak increases, while that of the NKMPO main phase peak decreases. This indicates a rise in impurity phase content and the destruction of the single-phase structure, which is required for efficient luminescence. In summary, in order to analyze the X-ray response range of NKMPO:Eu, PL results show that the optimal radio-photoluminescence doping concentration of Eu^3+^ is 0.14.

**Figure 2:**
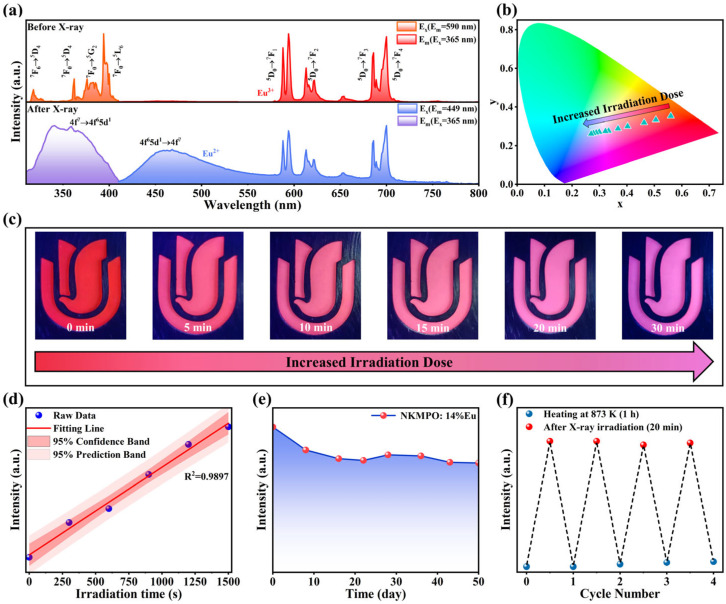
(**a**) PL and PLE spectra of NKMPO:Eu measured before and after X-ray irradiation (208 Gy). (**b**) CIE 1931 chromaticity diagram of NKMPO:0.14Eu as a function of irradiation dose (0–208 Gy). (**c**) Photographs of NKMPO:0.14Eu under 365 nm irradiation at different X-ray irradiation doses (6.933 Gy/min). (**d**) Linear response of radiation dose (6.933 Gy/min) and PL intensity (λ_ex_ = 365 nm, λ_em_ = 400–550 nm). (**e**) PL intensity (λ_ex_ = 365 nm, λ_em_ = 400–550 nm) of NKMPO:0.14Eu reading after different times (208 Gy). (**f**) X-ray information recording and erasure cycling test (λ_ex_ = 365 nm, λ_em_ = 400–550 nm).

As illustrated in Figure 2a, the PL and PLE spectra of NKMPO:Eu before and after X-ray irradiation at room temperature show changes in the luminescence center. A set of strong emission peaks was observed from 575 nm to 700 nm, corresponding to the ^5^D_0_→^7^F_J_ (J = 0,1,2,3 and 4) transitions of Eu^3+^. After X-ray irradiation, a broad emission band appeared at 450 nm, increasing in intensity with irradiation dose, while the emission peaks in the 575 nm to 700 nm range gradually decreased. The new emission band can be attributed to the 4f^6^5d^1^→4f^7^ transition of Eu^2+^ ions, which are formed through the reduction of Eu^3+^ during irradiation. As shown in Figure S6, PL decay life of NKMPO:0.14Eu was measured at room temperature. Before X-ray irradiation, the decay life of Eu^3+^ was 2.8879 ms. In contrast, the decay life of Eu^2+^ luminescence peak produced after X-ray irradiation was 2.3031 μs, which is three orders of magnitude shorter than that of the Eu^3+^ emission. The influence of Eu concentration on decay kinetics was further investigated. As shown in Figure S7, the luminescence lifetime of Eu^3+^ increases from 2.5887 ms to 2.9384 ms with rising Eu^3+^ concentration, deviating from conventional concentration quenching. This anomaly is attributed to trap-state formation at higher doping levels, which alters the decay pathway. Excitation energy is captured and stored in these traps, followed by slow release via radiative transitions, thereby extending the decay lifetime. The lifetime of Eu^2+^ emission decreases from 2.9479 μs to 2.0109 μs under combined effects of increasing doping concentration. This reduction suggests an elevated density of lattice defects such as $V_{Na}^{\prime }$ and $V_{O}^{∙∙}$ (oxygen vacancy), which likely accelerates non-radiative transitions of Eu^2+^ ions [44,46].

As shown in Figure 2b,c, increasing the irradiation dose from 0 to 30 min gradually changes the color of NKMPO:0.14Eu powder due to the increasing A_Eu_^2+^/A_Eu_^3+^ (Amount) ratio, shifting the color from red-orange to blue-purple. The corresponding CIE coordinates shift from (0.5604, 0.3526) to (0.2699, 0.2606). Figure S8 shows PL spectra of NKMPO:0.14Eu under various X-ray exposure durations, and the maximum limit of X-ray detection range of NKMPO:0.14Eu is about 200 Gy; this far exceeds the current commonly used commercial APG glass dose detectors [28,51]. Figure 2d shows a strong linear relationship between X-ray dose and emission intensity, exhibiting an excellent correlation (R^2^ = 0.9897) across the entire detection range; this suggests its potential application in the nondestructive detection of heavy and high-density materials [52,53]. These findings demonstrate that NKMPO:0.14Eu exhibits robust radio-photoluminescence properties, and X-ray-induced changes in its PL and PLE spectra make it a promising material for X-ray detection and imaging applications.

Typically, radiation detection materials store information about X-rays by trapping electrons and holes generated during X-ray exposure. The depth of these traps is an inherent material property that determines the material’s capacity to store X-ray information. As shown in Figure 2e, the X-ray storage capacity of NKMPO:0.14Eu at room temperature was evaluated. The PL intensity generated by X-ray irradiation shows minimal decay even after 50 days of storage. This is attributed to the deep traps that provide a high-energy barrier, allowing long-term signal retention. These results indicate that NKMPO:0.14Eu possesses excellent X-ray storage capability.

Most X-ray storage materials lose information at elevated temperatures, limiting their practical applications. To further evaluate the thermal stability of NKMPO:Eu, a batch of powders was irradiated with X-rays for the same duration and then subjected to heat treatment at various temperatures for 1 h. The fluorescence spectra obtained post-treatment are shown in Figure S9. From 293 K to 773 K, the PL intensity of NKMPO: 0.14Eu increases gradually. This enhancement is likely due to thermally assisted capture of residual free electrons by traps. However, when the temperature exceeds 773 K, the luminescence intensity of Eu^2+^ decreases. After heat treatment at 873 K for 1 h, the Eu^2+^ signal nearly disappears. This is because high thermal energy enables electrons and holes to escape from the RPL centers, and Eu^2+^ is oxidized back to Eu^3+^.

A series of X-ray irradiation and thermal erasure cycles were conducted to evaluate the reusability of NKMPO:0.14Eu, as shown in Figure 2f. After thermal treatment at 873 K, the emission intensity of Eu^2+^ returned to its original state, demonstrating the material’s capacity for repeated X-ray data storage. The experimental data show that the X-ray recording ability is maintained well in multiple cycles and is basically not affected by the number of cycles.

### 2.3. Photochromic Property

**Figure 3:**
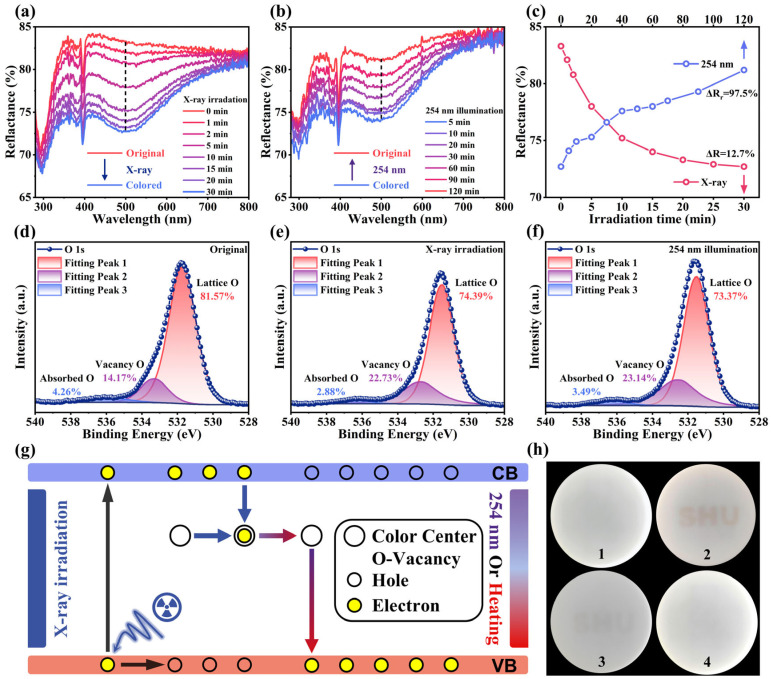
(**a**) Reflectivity spectra of fresh NKMPO:0.14Eu after various X-ray irradiation durations (6.933 Gy/min). (**b**) Reflectivity spectra of colored NKMPO:0.14Eu after various 254 nm illumination durations. (**c**) Reflectivity at 500 nm during photochromism and bleaching processes. (**d**–**f**) XPS spectra of the O element from the initial state, after X-ray coloring (208 Gy), and after 254 nm bleaching of the NKMPO:0.14Eu. (**g**) Schematic diagram of the mechanism of photochromic reaction. (**h**) Actual images before (1) and after (2) X-ray-induced photochromism (208 Gy), and at 30 min (3) and 100 min (4) after bleaching.

After X-ray irradiation, the color of the NKMPO:Eu changes from white to brown—an optical phenomenon known as photochromism [22]. Figure 3a shows the diffuse reflectance spectra (DRS) of photochromic samples after various durations of X-ray irradiation. For the unirradiated NKMPO:Eu, the narrow absorption peak centered at 395 nm is attributed to the 4f-4f transition of Eu^3+^, and the broad absorption peak centered at 296 nm is attributed to the charge transfer band (CTB). As X-ray exposure time increases, the absorption from the UV to visible region is enhanced, leading to increased discoloration. Color contrast is a key performance indicator for photochromic materials. To quantify the degree of coloration (ΔR) of the photochromic NKMPO:Eu, the ΔR located at 500 nm is expressed by the following formula [23,29,54]:

(2) $$\Delta R=(R_{0}-R_{i})∕R_{0}\times 100\%$$

where R_0_ and R_i_ represent DRS intensities of NKMPO:Eu before and after X-ray irradiation, respectively. As the irradiation time increased from 1 min to 30 min, the corresponding ΔR gradually changed from 1.4% to 12.7%, showing photochromic properties (Figure 3c). After 30 min, ΔR plateaued, suggesting saturation. Meanwhile, the relationship between diffuse reflectance intensity at 500 nm and the X-ray dose indicates that the photochromic degree of NKMPO:Eu exhibits a linear response to X-ray exposure.

In addition, reversible photochromism is an important parameter, as it reflects the repeatability of photochromic materials during use. To investigate this, the decolorization behavior of NKMPO:Eu under 254 nm UV light was studied. To minimize heat accumulation from prolonged high-intensity light source exposure, a low-power LED light source (1 W∙cm^−1^) was used. Figure 3b shows the DRS of NKMPO:Eu at various illumination durations. As illumination time increases, the absorption weakens, and its color gradually changes from brown to white. However, the bleaching effect is relatively limited; even with extended exposure, the sample does not fully return to its initial state. The degree of recovery, ΔR_r,_ at 500 nm is expressed as follows [23,29,54]:

(3) $$\Delta R_{r}=(R_{b}-R_{0})∕R_{0}\times 100\%$$

where R_0_ and R_b_ represent the DRS intensities of the NKMPO:Eu in its initial and bleached states, respectively. After 120 min of 254 nm LED exposure, the ΔR_r_ at 500 nm was calculated to be 97.7% (Figure 3c). Additionally, heat treatment proved to be a more effective bleaching method. Upon heating at 300 °C for 60 min, the sample fully reverted to its original color. As shown in Figure S10, ΔR_r_ reached 99.2%, indicating that thermal bleaching achieves a more complete recovery effect.

To better understand the properties of photochromism, we further investigated the underlying mechanism responsible for this phenomenon. In inorganic oxide photochromic materials, attention is typically focused on oxygen vacancies formed during the coloration process, as these defects can trap excited electrons and act as photochromic centers [23,55]. As shown in Figure 3d–f, the presence of oxygen vacancies in the NKMPO:Eu powder was confirmed by XPS. Recent studies have shown that since it is impossible for there to be photoelectron signals derived from missing oxygen atoms, the so-called oxygen vacancy signals observed in XPS tests are essentially just the hydroxyl groups that are incidentally adsorbed on the surface of the oxide, factors such as the content and state of oxygen vacancies affect their adsorption capacity [56]. This phenomenon is bound to occur under normal temperature and pressure conditions. However, we can still indirectly infer the changes in the content of oxygen vacancies by using the lattice oxygen signals and the signals from surface hydroxyl groups and water. Peaks at lower binding energies correspond to lattice oxygen, while peaks at higher binding energies are attributed to hydroxyl groups generated by the adsorption and dissociation of water molecules at oxygen vacancies (V_O_). Following X-ray irradiation, the signal associated with oxygen vacancies increased from 14.17% to 23.14%, while the proportion of lattice oxygen decreased. After subsequent 254 nm irradiation, the oxygen vacancy signal decreased slightly to 22.13%. This may be because X-ray irradiation in the XPS test also produced oxygen vacancy defects, narrowing the difference in signal intensity before and after 254 nm illumination. These changes in oxygen vacancy concentration were accompanied by the color change and bleaching effects, indicating a direct relationship between oxygen vacancies and photochromism [23,55]. To further illustrate this mechanism, a schematic diagram of the proposed photochromic process in NKMPO:Eu is presented in Figure 3g. Under X-ray irradiation, electrons in the valence band are excited to the conduction band (CB), and some of these electrons are subsequently captured by oxygen vacancy V_O_ defect. This forms photochromic centers that exhibit strong absorption in the visible region (300–800 nm), causing the NKMPO:Eu to appear brown. Upon exposure to 254 nm UV light, the trapped electrons are released and return to the valence band (VB), thereby restoring the original reflectivity and color—this is the bleaching process. Thermal stimulation can also achieve the same result. To visually demonstrate this effect, a template was used to write the message “SHU” on NKMPO:Eu@RTV film. Photographs of the color changes during the coloration and bleaching processes are shown in Figure 3h.

To further investigate the trap distribution related to oxygen vacancy defects, thermoluminescence (TL) measurements were conducted, as shown in Figure S12. The TL curve shows three distinct peaks, and the corresponding trap depth was calculated using the Hoogenstraaten method [29,55]:

(4) $$\Delta E=T_{m}/500$$

where ΔE is the trap depth, T_m_ (K) is the temperature corresponding to the TL peak (378 K), and the calculated defect energy corresponding to photochromism is 0.756 eV. Without strong external stimulation, carriers trapped in such shallow traps are released slowly. As shown in Figure S11, the written information “SHU” nearly disappeared after 7 days in darkness at room temperature, supporting the presence of shallow traps in NKMPO:Eu.

### 2.4. Photochromic and Radio-Photoluminescence Mechanism

**Figure 4:**
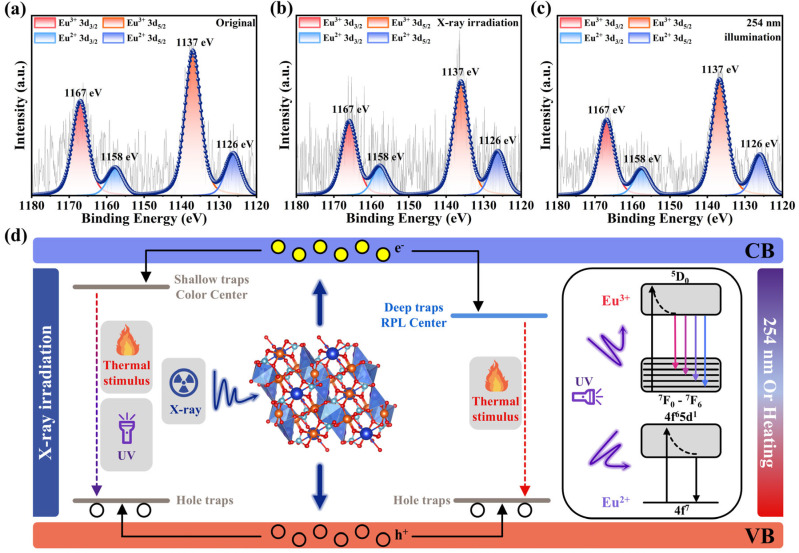
(**a**) XPS spectra of Eu element for NKMPO:0.14Eu before X-ray irradiation. (**b**) XPS spectra of Eu element for NKMPO:0.14Eu after X-ray irradiation (208 Gy). (**c**) XPS spectra of Eu element for NKMPO:0.14Eu after 254 nm irradiation. (**d**) Schematic of photochromism and radio-photoluminescence mechanism in NKMPO:Eu.

NKMPO:Eu exhibits dual-mode luminescence capability and shows strong potential for application in X-ray detection and imaging. To better understand and modulate its luminescence behavior, the relationship between the photoluminescence and radio-photoluminescence mechanisms of NKMPO:Eu was further investigated. First, to assess the effect of X-ray irradiation and 254 nm UV bleaching on the luminescent centers of NKMPO:0.14Eu, the valence state of europium was analyzed by XPS. The Eu3d XPS spectra of NKMPO:0.14Eu before X-ray irradiation, after X-ray irradiation, and after 254 nm UV bleaching are shown in Figure 4a–c. The signals at 1137 eV and 1167 eV correspond to Eu^3+^ 3d_5/2_ and Eu^3+^ 3d_3/2_, respectively, and the signals at 1126 eV and 1158 eV correspond to Eu^2+^ 3d_5/2_ and Eu^2+^ 3d_3/2_, respectively. Before X-ray irradiation, the Eu^3+^ (79.56%) signal is strong, and the Eu^2+^ (20.44%) signal is relatively weaker. After X-ray irradiation, the Eu^2+^ (25.83%) signal increases, and the Eu^3+^ (74.17%) signal decreases, indicating that the luminescence center gradually transforms from Eu^3+^ to Eu^2+^. Compared with the X-ray-irradiated state, the relative signal intensities of Eu^3+^ (75.92%) and Eu^2+^ (24.08%) remain nearly unchanged after 254 nm UV bleaching. The Eu^2+^ signal observed in the “Original” XPS spectra is attributed to the reduction of Eu^3+^ induced by the high-flux X-ray during measurement. Moreover, this instrumental effect partially masks the irradiation-driven valence change, consequently narrowing the difference in signal intensity of Eu^2+^/Eu^3+^ before and after X-ray irradiation.

Meanwhile, as shown in Figure S12, the TL test results indicate that one shallow trap and two deep traps were formed during X-ray irradiation, and the intensities of all traps increased with longer irradiation times. When the photochromic centers were exposed to 254 nm UV light, the intensity of the shallow trap decreased rapidly, while changes in deep trap intensity were relatively minimal. This suggests that the shallow trap is associated with the photochromic effect, while the deep traps are related to radio-photoluminescence. The energy from 254 nm UV light is sufficient to release electrons trapped in oxygen vacancies (photochromic centers) back into the VB. However, this energy is insufficient to release electrons captured in deep traps associated with radio-photoluminescence centers. In other words, the photochromic bleaching process has minimal effect on radiative photoluminescence intensity. This conclusion is supported by PL test results. As shown in Figure S13, after 254 nm UV bleaching, the X-ray-induced radio-photoluminescence intensity remained largely unchanged, confirming that 254 nm UV light has minimal impact on the radio-photoluminescence mechanism. The slight increase in Eu^2+^ emission intensity could be attributed to UV-induced electron capture by Eu^3+^.

In summary, we propose a mechanism for photochromism and radio-photoluminescence in NKMPO:Eu arising from its interaction with high-energy X-ray photons, as illustrated in Figure 4d.

(5) $$O^{2-}+h\nu \to V_{O}^{∙∙}+2e^{-}$$

(6) $${Eu}_{Na}^{∙∙}+e^{-}\to {Eu}_{Na}^{∙}\;or\;{Eu}^{3+}+e^{-}\to {Eu}^{2+}$$

(7) $$V_{O}^{∙∙}+e^{-}\to V_{O}^{∙}$$

During the sintering of NKMPO:Eu, high temperatures and Eu doping promote the formation of cation vacancies (Na, K, Mg) and oxygen vacancies within the lattice. Oxygen vacancies act as charge compensators, while cation and oxygen vacancies function as hole traps (H-traps) and electron traps (E-traps), respectively. When the material is exposed to X-ray irradiation, on the one hand, the lattice oxygen (O^2−^) in the crystal is ionized to release electrons and generate oxygen vacancies ($V_{O}^{∙∙}$) (Equation (5)). On the other hand, high-energy electron–hole pairs are generated, triggering a chain reaction: some excited electrons are captured by trivalent ions (${Eu}_{Na}^{∙∙}$ or ${Eu}^{3+}$) and reduced to divalent ions (${Eu}_{Na}^{∙}$ or ${Eu}^{2+}$) to form a radio-photoluminescent center (Equation (6)), while some electrons are captured by oxygen vacancies to form a photochromic center ($V_{O}^{∙}$) (Equation (7)), and the holes are captured by cation vacancy traps. In the photochromic centers, both UV light and thermal stimulation can supply enough energy to release trapped electrons back to the VB, enabling the reversibility of photochromism. However, for radio-photoluminescence centers, the energy required to release trapped electrons is higher and cannot be easily provided by conventional UV light. In this case, thermal treatment serves as a straightforward method to restore luminescence, supporting the reusability of radio-photoluminescent materials.

### 2.5. Optical Information Storage and Information Encryption Applications

**Figure 5:**
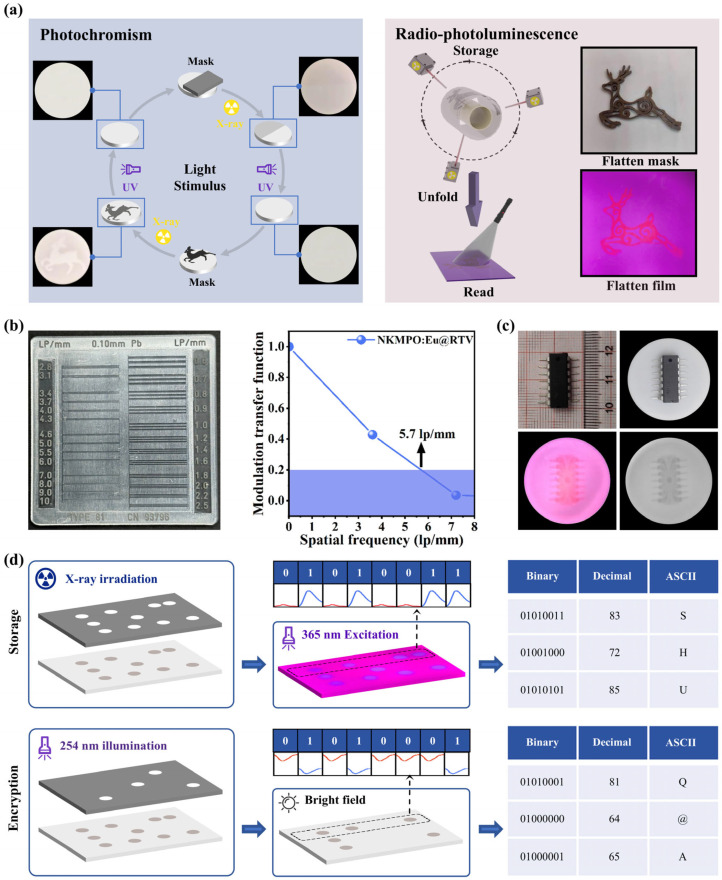
(**a**) Photochromism and radio-photoluminescence images of the NKMPO:Eu@RTV film. (**b**) Standard line pair card (**left**) and gray value modulation (**right**). (**c**) Detailed X-ray images of CMOS captured with a color camera and a black-and-white camera. (**d**) Information encryption application based on the mutual modulation of photochromism and radio-photoluminescence.

Inspired by the excellent dual-modal luminescence performance of NKMPO:Eu, we explored its application in optical information storage and anti-counterfeiting. Figure 5a illustrates the multimodal luminescence properties of NKMPO:Eu including both photochromism and radio-photoluminescence—which offer versatile strategies for optical information writing and reading.

First, photochromic images were generated using NKMPO:Eu@RTV film. Repeated cycles of writing and erasing demonstrated its capacity for recording and retrieving X-ray information under visible light and delayed time conditions. Second, we evaluated the flexible imaging performance under delayed conditions using radio-photoluminescence. To demonstrate this, NKMPO:Eu powder was embedded into room-temperature vulcanized (RTV) silicone to fabricate a flexible film for X-ray imaging. Using a time-delay mode, high-quality imaging of curved surfaces was achieved, wherein the storage and reading steps were conducted separately. As shown in Figure 5b, using the radio-photoluminescence property with a standard line pair card, the NKMPO:Eu@RTV film exhibits an excellent spatial resolution of 5.7 lp/mm. Based on simple theoretical calculations, the upper limit of the resolution of the detector we use is approximately 6 lp/mm. Therefore, this result has reached the best resolution capability under the test conditions using the detector. In Figure 5c, during X-ray irradiation, varying material densities beneath different interfaces (COMS) resulted in differential X-ray attenuation. Consequently, the radiation dose received by the flexible film varied, leading to different photoluminescence intensities under UV excitation. This technology has potential applications in areas such as security inspection and industrial defect detection—for example, in identifying gaps, misalignments, or circuit disconnections in electronic components.

Furthermore, we investigated an information encryption application based on the combined modulation of photochromism and radio-photoluminescence, as illustrated in Figure 5d. Thin films of NKMPO:Eu@RTV were irradiated under a 3 × 8 lattice template using X-rays for 20 min, resulting in a color change from white to brown in the exposed regions. Under 365 nm excitation, variations in the luminescence intensities of Eu^2+^ and Eu^3+^ were used to represent binary codes, with Eu^2+^ corresponding to “0” and Eu^3+^ to “1”. For example, the dot sequence in the first row produced the binary line “01010011”. This binary output was then converted into a decimal format and decoded using the ASCII table, revealing the encrypted message “SHU”. Next, the sample was selectively bleached with 254 nm UV light for further encoding. When observed under ambient light, the different reflectance levels again corresponded to binary values “0” and “1”. As shown in the first row, the derived binary line was “01010001”, which, upon conversion, yielded the decrypted message “Q@A”—a completely different result from that obtained under 365 nm light. This demonstrates the use of dual-modality (photochromism and radio-photoluminescence) in NKMPO:Eu for recording, encrypting, and inducing errors in optical information, highlighting its potential for secure, multimodal luminescence-based data storage and encryption.

## 3. Materials and Methods

### 3.1. Material Synthesis

The NKMPO:Eu powders were synthesized using a high-temperature solid-state reaction method. Firstly, raw materials sodium bicarbonate (99.5%), potassium bicarbonate (99.5%), magnesium oxide (98%), ammonium dihydrogen phosphate (99%), and high purity Eu_2_O_3_ (99.99%) were ground in an agate mortar in stoichiometric ratio. After grinding evenly, the mixture was heated in the air at 500 °C in the Muffle furnace for 4 h. The cooled sample was then re-ground in an agate mortar and then heated in the Muffle furnace to 950 °C for 6 h. Finally, the cooled sample is re-ground into a powder for further analysis. The raw materials are sourced from Shanghai Aladdin Biochemical Technology Co., Ltd. (Shanghai, China) and all ingredients are used as is. The chemical reactions for NKMPO:Eu are as follows:

$$(3-3x)NaHCO_{3}(s)+KHCO_{3}(s)+7MgO(s)+6NH_{4}H_{2}{PO}_{4}(s)+\frac{x}{2}{Eu}_{2}O_{3}(s)⟶$$

$${Na}_{3(1-x)}K{Mg}_{7}(PO_{4}{)}_{6}:xEu(s)+6{NH}_{3}(g)↑+(11-\frac{3}{2}x)H_{2}O(g)↑+(4-3x){CO}_{2}(g)↑$$

NKMPO:Eu powder fully ground in agate mortar was passed through different mesh screens to obtain powder samples of different particle sizes. The powder sample is mixed with RTV glue at a 1:1 mass ratio, and the RTV glue is obtained by mixing 9:1 mass ratio of colloidal and curing agent. The obtained colloidal sample is poured on a plastic Petri dish and left for 30 min in 80 °C drying oven before being taken out to obtain NKMPO:Eu@RTV composite film. The RTV glue is from Wacker Chemicals (China) Co., Ltd. (Shanghai, China), and the Petri dishes are from Changde Beekman Biotechnology Co., Ltd. (Changde, China), and are used as is.

### 3.2. Characterization

The X-ray diffraction (XRD) patterns of the prepared samples were collected on a powder X-ray diffractometer (Bruker D2 Phaser, Bruker AXS GmbH, Karlsruhe, Germany) with Cu Kα radiation (λ = 1.54178 Å) at in a step-scanning mode. The structure information was analyzed by the Rietveld method using General Structure Analysis System (GSAS-II) software, release 5817. Scanning electron microscopy (SEM) images were obtained using a Gemini-300 (Carl Zeiss AG, Oberkochen, Germany), using secondary electron (SE) modes at an electron-beam voltage of 5 kV, and the element distribution of the sample was identified by energy-dispersive X-ray spectroscopy (EDS) combined with a scanning electron microscope. The diffuse reflectance spectra (DRS) were measured on a U-3900 UV-Vis spectrophotometer (Hitachi High-Tech Corporation, Tokyo, Japan), using BaSO_4_ as the reference. Photoluminescence properties were characterized at 298 K using an FLS1000 spectrofluorometer (Edinburgh Instruments Ltd., Livingston, UK). For steady-state measurements including excitation and emission spectra, a continuous xenon lamp served as the excitation source and 395 nm filter was used on the excitation port side. For time-resolved decay measurements, the excitation source was substituted with a μF2 microsecond flash lamp (Edinburgh Instruments, UK) to enable precise registration of microsecond-scale lifetimes. The signals were processed using time-correlated single photon counting (TCSPC) electronics, with data acquisition continuing until 10,000 counts accumulated at the emission peak. X-ray generator (NDT160K200205MR, Shenzhen Xinjiechu Machinery Technology Company, Shenzhen, China) as the radiation source, the current and voltage were adjusted to obtain different dose rates; its tube voltage is 40–150 kV, and the current is 200–1000 μA. When the voltage was set at 150 kV and the current was 1000 μA, the dose rate at the window was 6.933 Gy/min. X-ray photoelectron spectroscopy (XPS) measurements were performed on a Thermo Scientific K-Alpha model spectrometer (Thermo Fisher Scientific, Waltham, MA, USA) with monochromatic Al Kα radiation (1486.6 eV). Thermoluminescence (TL) spectra were acquired using a TOSL-3DS model spectrometer (Radiascan Technology Co., Ltd., Guangzhou, China) equipped with a PMT detector.

## 4. Conclusions

In conclusion, we developed a dual-modal luminescent NKMPO:Eu that integrates photochromism and radio-photoluminescence within a single material. Upon X-ray irradiation, oxygen vacancy-related color centers form, resulting in a photochromic effect that correlates well with radiation dose, changing the material’s color from white to brown. Both optical and thermal stimulation were effective in achieving bleaching. Additionally, the material exhibited radio-photoluminescence due to the conversion of Eu^3+^ to Eu^2+^, showing a strong linear response (R^2^ = 0.9897) across high radiation doses (maximum dose > 200 Gy), excellent information storage capability (>50 days), and good reusability. Importantly, UV-induced bleaching had minimal impact on the intensity of radio-photoluminescence, suggesting potential applications in optical information encryption and anti-counterfeiting. Finally, an RTV-based flexible film incorporating NKMPO:Eu demonstrated practical application prospects in X-ray detection and imaging, including multi-mode display, non-destructive testing, and secure data storage.

## Data Availability

The original contributions presented in the study are included in the article/Supplementary Materials; further inquiries can be directed to the corresponding author.

## Supplementary Materials

The following supporting information can be downloaded at: https://www.mdpi.com/article/10.3390/molecules30173495/s1, Figure S1: (a) Schematic diagram of NKMPO:Eu (Na_3_KMg_7_(PO_4_)_6_:Eu) powder preparation. Powder synthesis via two-stage heat treatment (primary/secondary heating) with intermediate grinding. (b) Schematic diagram of NKMPO:Eu@RTV film preparation. Film fabrication by mixing NKMPO:Eu phosphor with RTV glue at [1:1] mass ratio, followed by curing; Figure S2: The illustration of crystal structure of NKMPO; Table S1: Ion radius percentage difference (D_r_) between Eu^3+^ and cations (Na^+^, K^+^, Mg^2+^) in the host; Figure S3: XRD of NKMPO:0.02Eu synthesized under different temperature conditions; Table S2: Rietveld refinement parameters of X-ray diffraction of NKMPO:*x*Eu (*x* = 0.02–0.20); Figure S4: PL spectra of NKMPO:Eu synthesized under different temperature conditions (λ_ex_ = 365 nm). Figure S5: PL spectra and PL intensty at 612 nm of NKMPO:*x*Eu (*x* = 0.02, 0.05, 0.08, 0.10, 0.12, 0.13, 0.14, 0.15, 0.18, and 0.20) (λ_ex_ = 365 nm); Figure S6: Time-resolved decay time curve of NKMPO:0.14Eu before (λ_ex_ = 365 nm, λ_em_ = 612 nm) and after X-ray irradiation (λ_ex_ = 365 nm, λ_em_ = 459 nm/612 nm) under the microsecond lamp at room temperature (298 K); Figure S7: Time-resolved decay time curve of NKMPO:xEu (*x* = 0.02 - 0.20) before (λ_ex_ = 365 nm, λ_em_ = 612 nm) and after X-ray irradiation (λ_ex_ = 365 nm, λ_em_ = 459 nm) under the micro/nanosecond lamp at room temperature (298 K); Figure S8: PL spectra of NKMPO:0.14Eu with different doses (λ_ex_ = 365 nm); Figure S9: PL intensity (λ_ex_ = 365 nm) at 449 nm of NKMPO:0.14Eu after different heat treatments; Figure S10: Reflectivity spectra of colored NKMPO:0.14Eu after various heat treatments; Figure S11: Color change photographs captured after different delay times at room temperature (298 K); Figure S12: TL curves of NKMPO:Eu with different X-ray dose (6.933 Gy/min) and different 254 nm illumination dose; Figure S13: PL spectra measured before and after the bleaching process.
