# Supplementary material for: High-Performance X-Ray Detection and Optical Information Storage via Dual-Mode Luminescent Modulation in Na3KMg7(PO4)6:Eu
Source: Molecules. 2025 Aug 26;30(17):3495. doi: 10.3390/molecules30173495 (PMC12430156; doi:10.3390/molecules30173495)
Supplement: Supplementary file 1 [file molecules-30-03495-s001.zip › Supporting Information.pdf]

Supporting Information

High-Performance X-ray Detection and Optical Information Storage via Dual-Mode Luminescent Modulation in $Na_3KMg_7(PO_4)_6$:Eu

Yanshuo Han [1,†], Yucheng Li [2,†], Xue Yang [1], Yibo Hu [1], Yuandong Ning [1], Meng Gu [1,3], Guibin Zhai [1,4], Sihan Yang [1], Jingkun Chen [1], Naixin Li [1], Kuan Ren [4], Jingtai Zhao [3] and Qianli Li [1,*]

[1] State Key Laboratory of Materials for Advanced Nuclear Energy, School of Materials Science and Engineering, Shanghai University, Shanghai 200444, China
[2] Shanghai Frontier Base of Intelligent Optoelectronics and Perception, Institute of Optoelectronics, Fudan University, Shanghai 200433, China
[3] Guangxi Key Laboratory of Information Materials, Guilin University of Electronic Technology, Guilin 541004, China
[4] Laser Fusion Research Center, China Academy of Engineering Physics, Mianyang 621050, China
* Correspondence: liqianli@shu.edu.cn
† These authors contributed equally to this work.

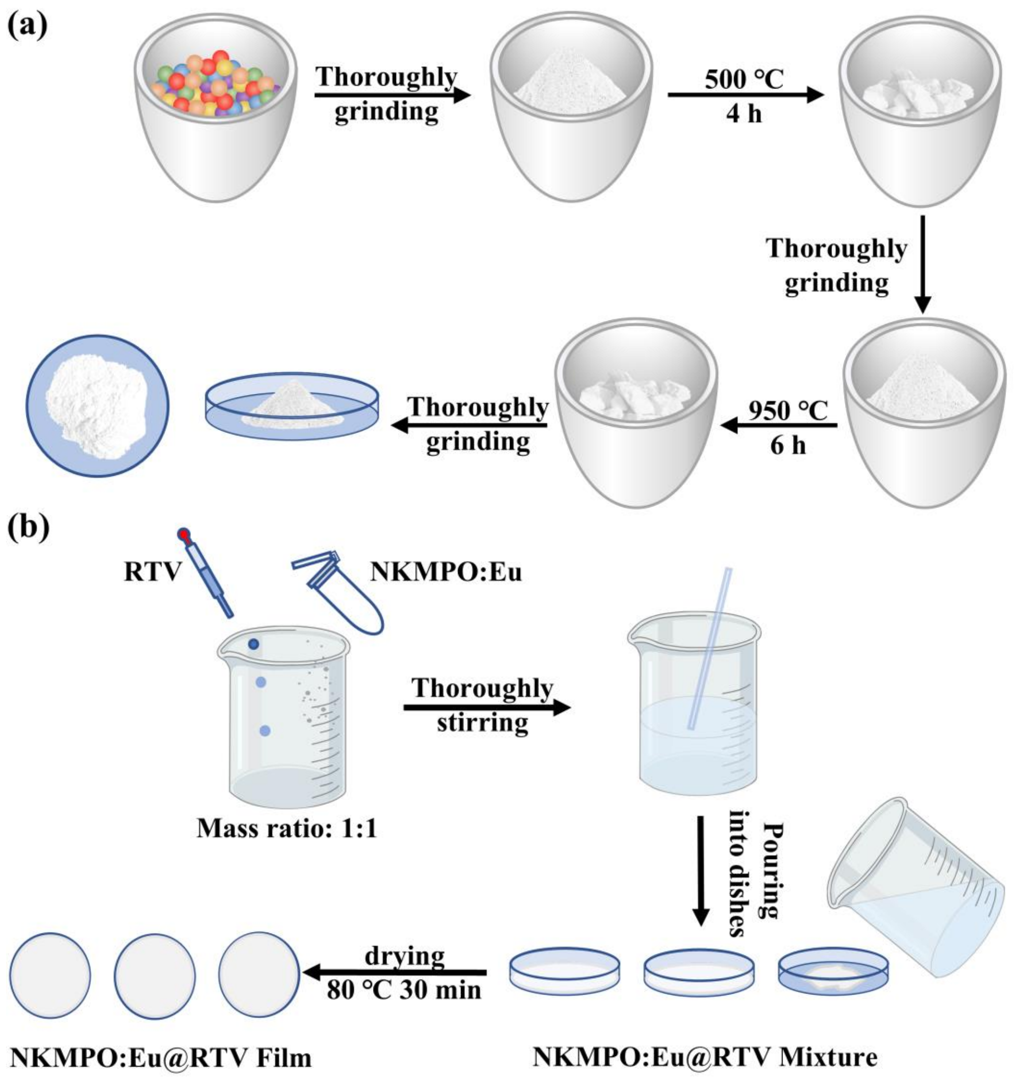

Figure S1. (a) Schematic diagram of NKMPO:Eu ($Na_3KMg_7(PO_4)_6$:Eu) powder preparation. Powder synthesis via two-stage heat treatment (primary/secondary heating) with intermediate grinding. (b) Schematic diagram of NKMPO:Eu@RTV film preparation. Film fabrication by mixing NKMPO:Eu phosphor with RTV glue at [1:1] mass ratio, followed by curing.

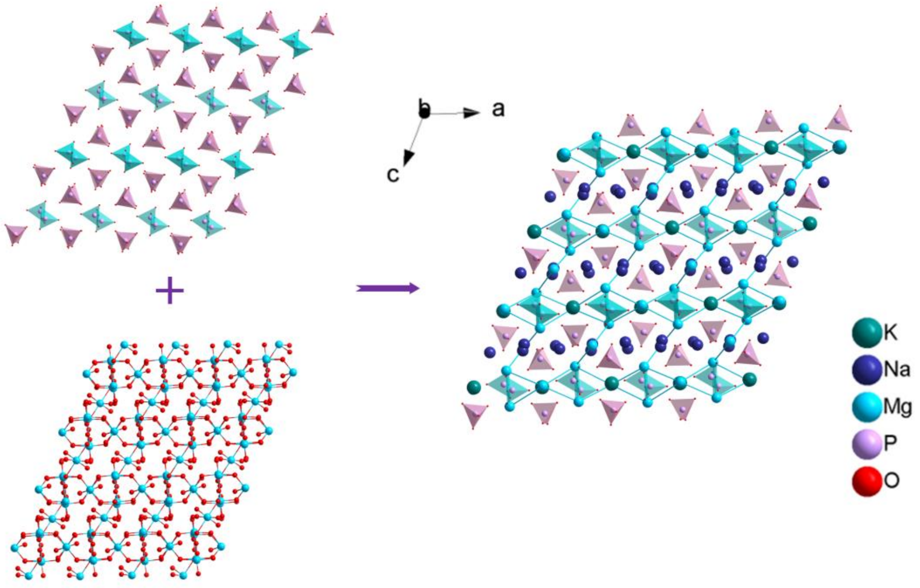

Figure S2. The illustration of crystal structure of NKMPO.

Ion	Coordination	Ionic radii (Å)	D_r
K^+	12(Standard)	1.64	25.61%
Na^+	8(Standard)	1.18	9.32%
Na^+	6(Standard)	1.02	6.86%
Mg^{2+}	6(Standard)	0.72	31.94%
Mg^{2+}	5(Standard)	0.66	34.85%
Eu^{3+}	5(Non-standard)	0.89	
Eu^{3+}	6(Standard)	0.95	
Eu^{3+}	8(Standard)	1.07	
Eu^{3+}	12(Non-standard)	1.22	

Table S1. Ion radius percentage difference (D_r) between Eu^{3+} and cations (Na^+, K^+, Mg^{2+}) in the host.

$$D_r = \frac{|R_m(CN) - R_d(CN)|}{R_m(CN)}$$

Where D_r is the ion radius percentage difference, CN is the coordination number, $R_m(CN)$ is the radius of the cation in the host, and $R_d(CN)$ is the radius of Eu^{3+} ion. Values labeled "Standard" derive from Shannon' s crystallographic compilation, providing reference radii for common coordination numbers. Values labeled "Non-standard" originate from machine learning predictions.

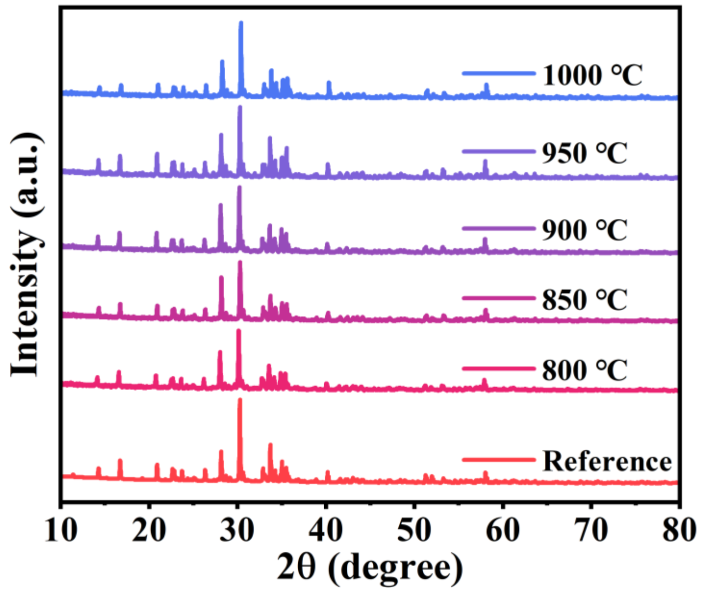

Figure S3. XRD of NKMPO:0.02Eu synthesized under different temperature conditions.

.

Compound	$Na_3KMg_7(PO_4)_6$:*x*Eu		T/°C	25	Space group	C2/c Monoclinic
**Concentration**	**0.02**	**0.05**	**0.10**	**0.14**	**0.20**	**Reference**
***a*/nm**	1.272935	1.272999	1.273269	1.273758	1.273835	1.27239
**b/nm**	1.066808	1.066963	1.067153	1.066791	1.066659	1.06584
***c*/nm**	1.544977	1.546937	1.546953	1.547406	1.548477	1.5473
**β/(°)**	112.849	112.841	112.859	112.863	112.877	112.921
**Volume/nm^3**	1.933407	1.936357	1.936883	1.937472	1.938492	1.932707
Z	4	4	4	4	4	4
**Occupancy Eu-Na(1)**	0.0147	0.0218	0.0360	0.0465	0.0850	
**Occupancy Eu-Na(2)**	0.0053	0.0282	0.0640	0.0935	0.1150	
**R_{wp}/%**	6.268	5.850	6.405	9.102	6.882	
**χ^2**	0.82	0.72	0.87	1.80	1.05	

Table S2. Rietveld refinement parameters of X-ray diffraction of NKMPO:*x*Eu (*x* = 0.02 - 0.20).

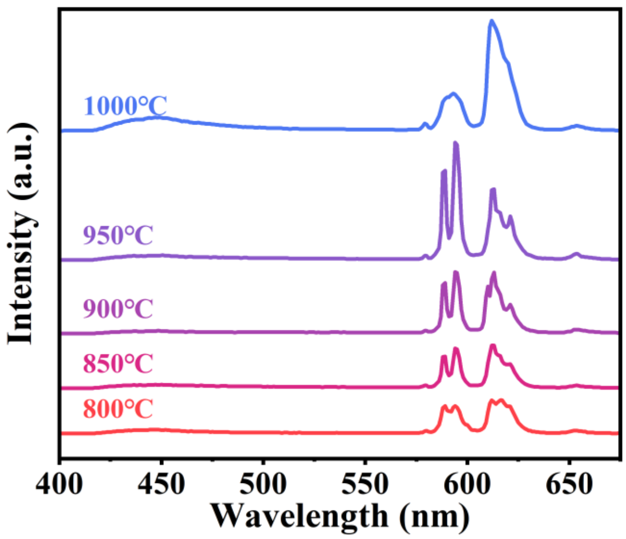

Figure S4. PL spectra of NKMPO:Eu synthesized under different temperature conditions (λ_{ex} = 365 nm).

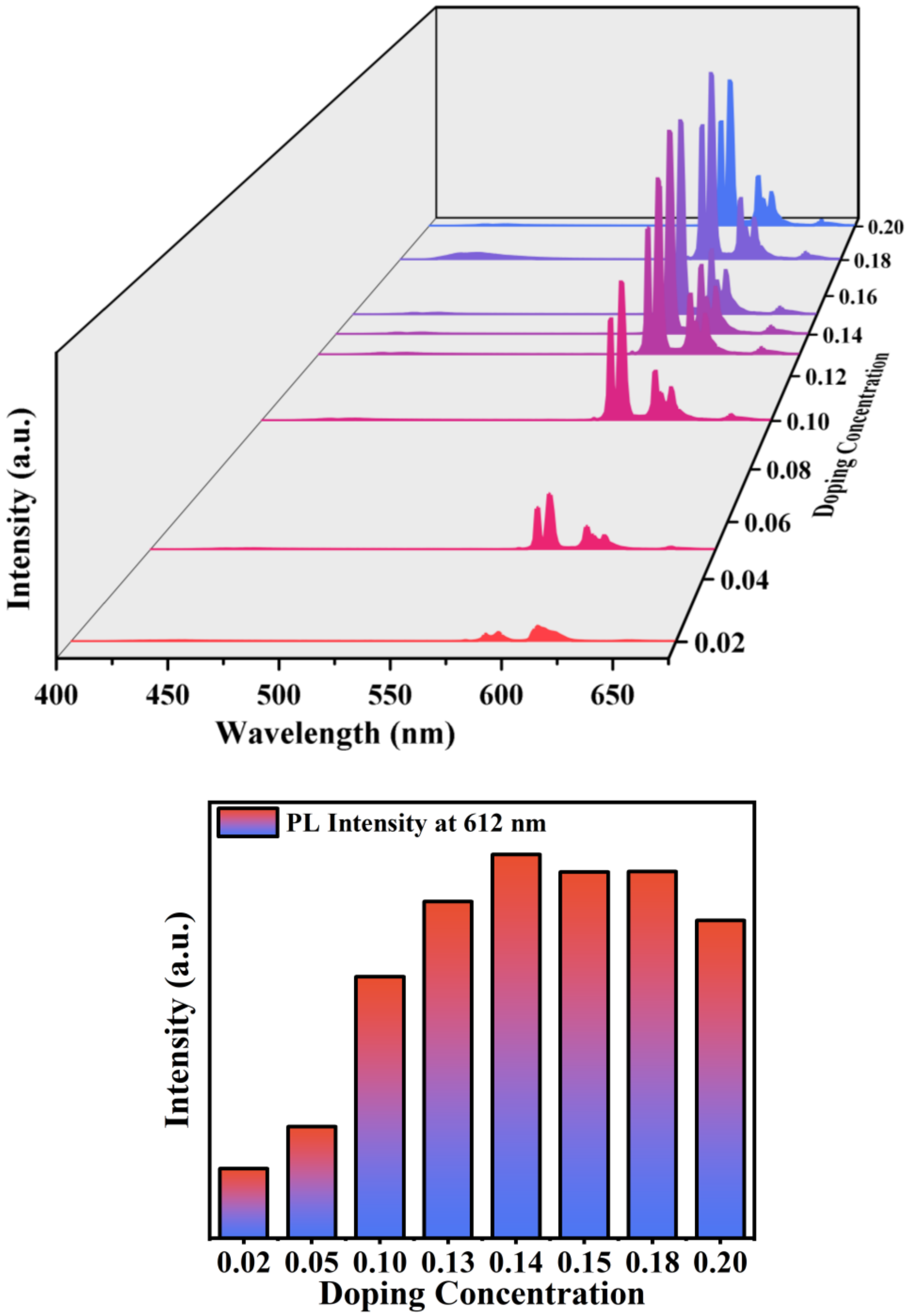

Figure S5. PL spectra and PL intensty at 612 nm of NKMPO:xEu (x = 0.02, 0.05, 0.08, 0.10, 0.12, 0.13, 0.14, 0.15, 0.18, and 0.20) (λ_{ex} = 365 nm).

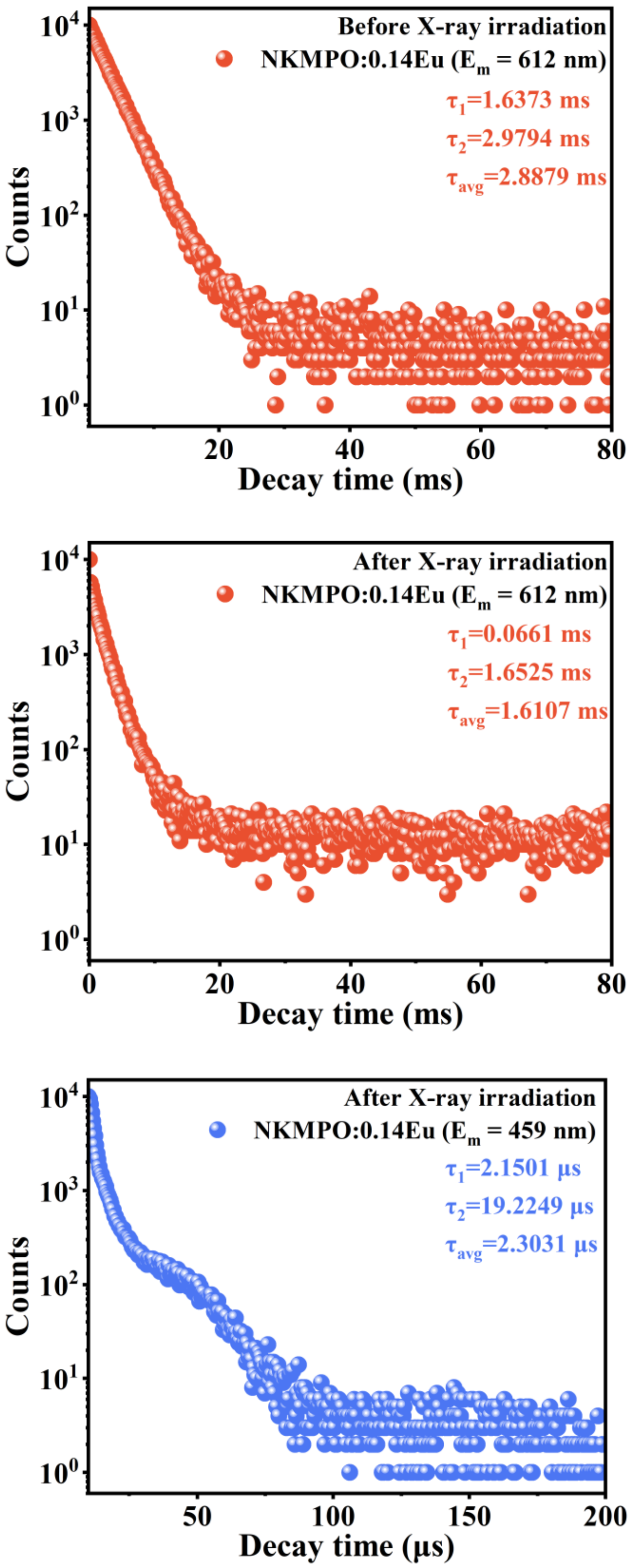

Figure S6 Time-resolved decay time curve of NKMPO:0.14Eu before (λ_{ex} = 365 nm, λ_{em} = 612 nm) and after X-ray irradiation (λ_{ex} = 365 nm, λ_{em} = 459 nm/612 nm) under the microsecond lamp at room temperature (298 K).

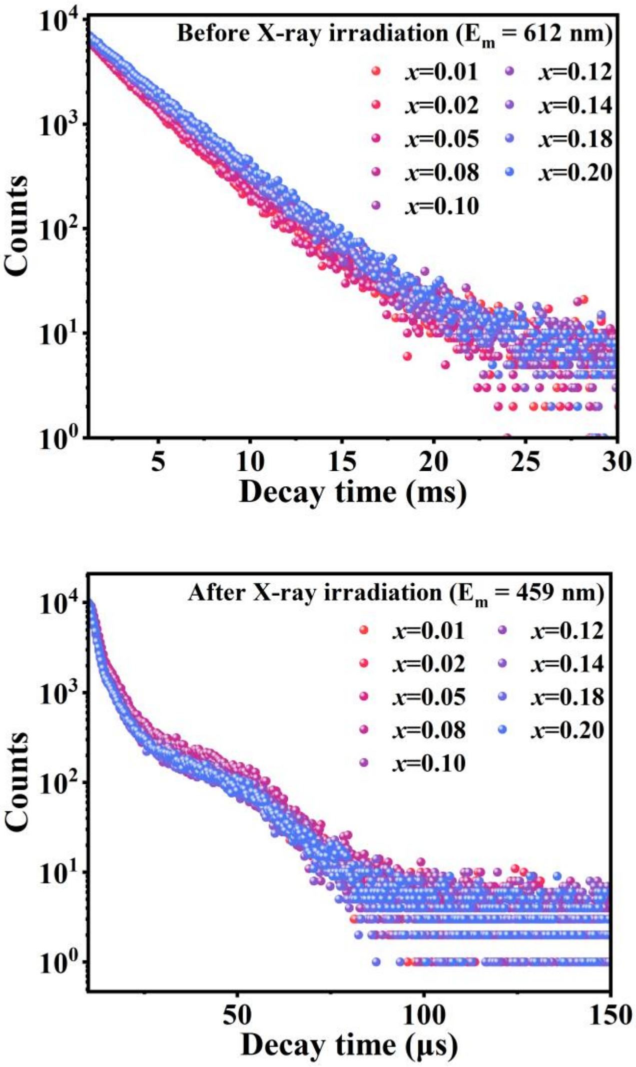

Figure S7 Time-resolved decay time curve of NKMPO:xEu (x = 0.02 - 0.20) before (λ_{ex} = 365 nm, λ_{em} = 612 nm) and after X-ray irradiation (λ_{ex} = 365 nm, λ_{em} = 459 nm) under the micro/nanosecond lamp at room temperature (298 K).

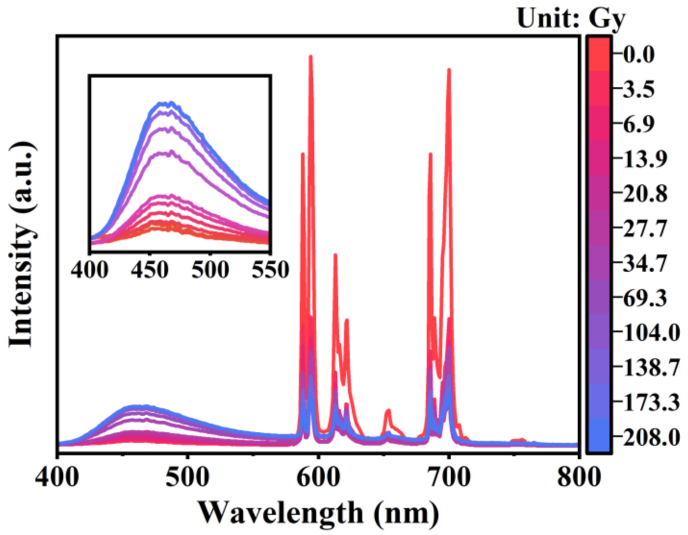

Figure S8. PL spectra of NKMPO:0.14Eu with different doses (λ_{ex} = 365 nm).

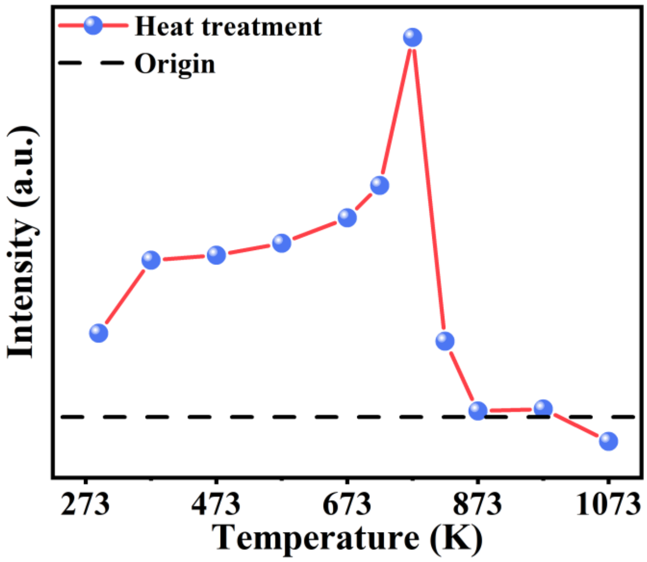

Figure S9. PL intensity (λ_{ex} = 365 nm) at 449 nm of NKMPO:0.14Eu after different heat treatments.

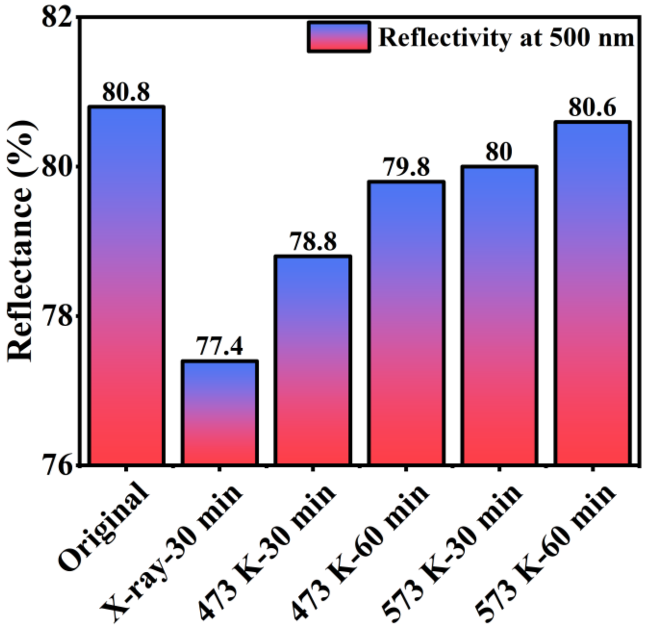

Figure S10. Reflectivity spectra of colored NKMPO:0.14Eu after various heat treatments.

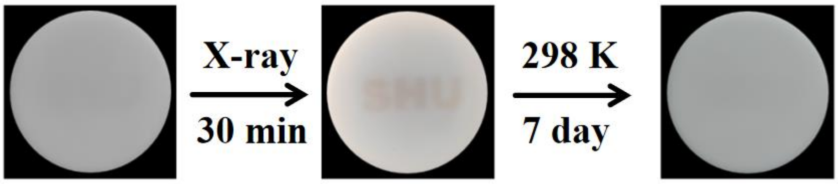

Figure S11. Color change photographs captured after different delay times at room temperature (298 K).

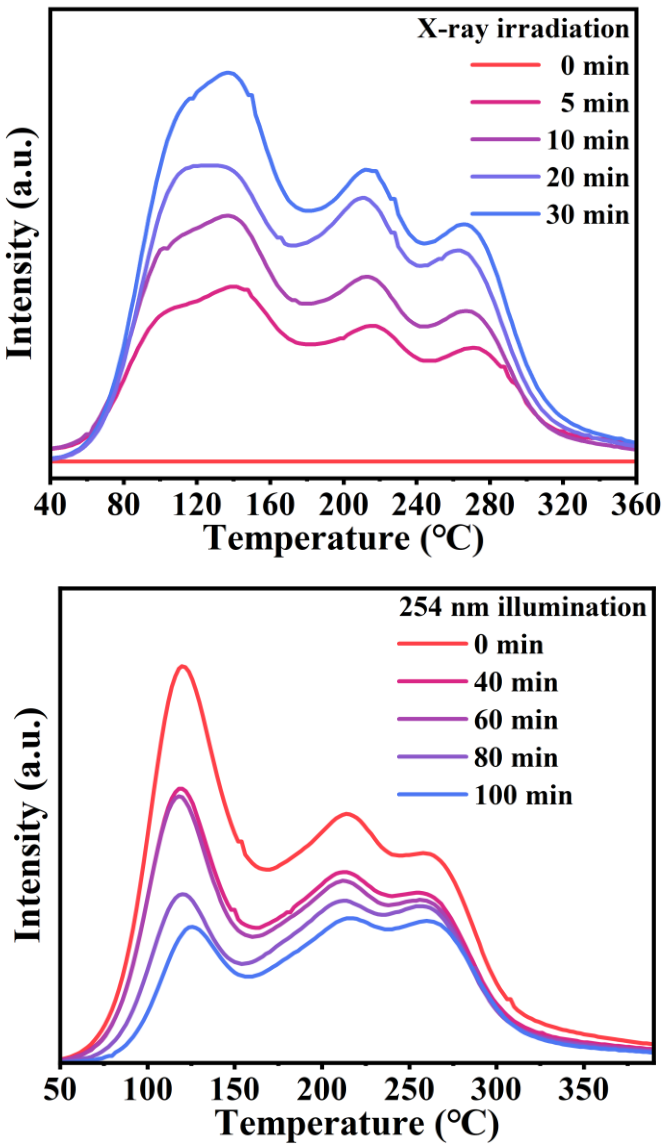

Figure S12. TL curves of NKMPO:Eu with different X-ray dose (6.933 Gy/min) and different 254 nm illumination dose.

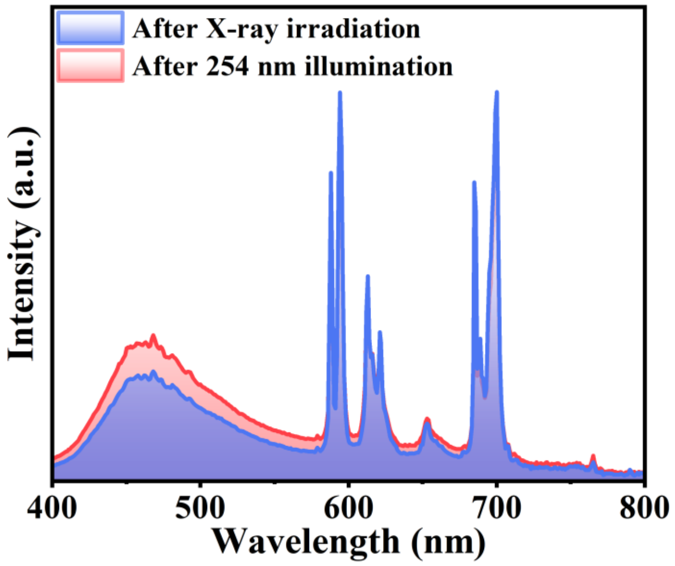

Figure S13. PL spectra measured before and after the bleaching process.
